# Hepatitis B virus inhibition in mice by lentiviral vector mediated short hairpin RNA

**DOI:** 10.1186/1471-230X-9-73

**Published:** 2009-10-06

**Authors:** Lei Deng, Guoqiang Li, Lisen Xi, Aihong Yin, Yun Gao, Wei You, Xuehao Wang, Beicheng Sun

**Affiliations:** 1 Liver Transplantation Center of the First Affiliated Hospital, Nanjing Medical University, 300 Guangzhou Road, Nanjing, Jiangsu 210029, PR China

## Abstract

**Background:**

Chronic hepatitis B virus (HBV) infection is an important cause of cirrhosis and hepatocellular carcinoma. The major challenges for current therapies are the low efficacy of current drugs and the occurrence of drug resistant HBV mutations. RNA interference (RNAi) of virus-specific genes offers the possibility of developing a new anti-HBV therapy. Recent reports have shown that lentiviral vectors based on HIV-1 are promising gene delivery vehicles due to their ability to integrate transgenes into non-dividing cells. Herein, a lentivirus-based RNAi system was developed to drive expression and delivery of HBV-specific short hairpin RNA (shRNA) in a mouse model for HBV replication.

**Methods:**

Hepatitis B surface antigen (HBsAg) and hepatitis B e antigen (HBeAg) in the sera of the mice were analyzed by quantitative sandwich enzyme linked immunosorbent assay (ELISA) technique, hepatitis B core antigen (HBcAg) and HBsAg in the livers of the mice were detected by immunohistochemical assay, HBV DNA and HBV mRNA were measured by fluorogenic quantitative polymerase chain reaction (FQ-PCR) and quantitative real-time PCR respectively.

**Results:**

Co-injection of HBV plasmids together with the lentivirus targeting HBV shRNA induced an RNAi response. Secreted HBsAg was reduced by 89% in mouse serum, and HBeAg was also significantly inhibited, immunohistochemical detection of HBcAg or HBsAg in the liver tissues also revealed substantial reduction. Lentiviral mediated shRNA caused a significant suppression in the levels of viral mRNA and DNA synthesis compared to the control group.

**Conclusion:**

Lentivirus-based RNAi can be used to suppress HBV replication in vivo, it might become a potential therapeutic strategy for treating HBV and other viral infections.

## Background

Hepatitis B virus (HBV) infection is still a worldwide health problem. There are an estimated 400 million chronic HBV infected patients worldwide, especially in China, where the infection rate is even as high as 9.8% and over one million people die of liver failure or HBV-associated liver cirrhosis and hepatocellular carcinoma (HCC) annually[1]. Attempts at treatment of chronic infections have had only limited success. Because of the low efficacy or side effects of current drugs and the occurrence of drug resistant HBV mutations, only about 20% of the patients benefit from combination therapy with interferon-α and nucleoside analogues such as lamivudine, entecavir and adefovir dipivoxil [2-6]. Therefore, there is an urgent need to develop a new therapeutic strategy that effectively inhibits HBV replication.

The HBV virion comprises an envelope and a nucleocapsid containing a circular, partially double-stranded 3.2-kb DNA, which replicates via an RNA intermediate[7]. Recent studies have shown that RNA interference (RNAi), which can be induced in mammalian cells by short hairpin RNAs (shRNAs), is an evolutionarily conserved surveillance mechanism that responds to double-stranded RNAs (dsRNAs) by sequence-specific post-transcriptional silencing of homologous genes[8]. It is most important for the processing of primary miRs, which have important functions for the regulation of gene expression, and thus inhibits the replication of HBV [9-11]. Synthetic shRNA duplexes and plasmid-derived shRNAs have been shown to inhibit HIV-1 infection and replication by specifically degrading HIV genomic RNA [12-17]. It has also been shown that shRNA targeting HCV genomic RNA can inhibit HCV replication[15,18-20]. However, synthetic shRNAs transfected into target cells can only be retained for a few days in cells and will be diluted after several generations of cell division. In many cases, a long-term effect of RNAi is required. Thus, the selection of transfer vectors for RNAi has been a focus for RNAi research. To solve this problem, different alternatively used transfer vehicles, such as adeno-associated viral vectors(AAV) and adenoviral vectors were also explored for *in vivo *delivery of shRNA against HBV infection [21]. Yet, both of them have prominent drawbacks. For example, recombinant AAV will be lost eventually as it is episomal, and limits their application. Retroviral vectors derived from murine leukemia virus (MuLV) are favourable in gene delivery for their efficient integration into the genome of the target cells and accompanying expression of the transgenes. However, these vectors require cell division for efficient gene transfer[22,23]. To circumvent this problem, vector systems based on the lentivirus genus of retroviruses, which includes human immunodeficiency virus (HIV), are being developed[24]. These lentiviral vectors are able to transduce non-dividing cells with sustained long-term expression of the genes. The majority of target cell types for gene therapy are non-dividing or slowly dividing, such as hepatocytes[25]. Therefore, these properties of RNAi vector systems based on HIV or other lentiviruses open up a possibility of efficiently controlling replication processes of infectious viruses such as HBV, and have the potential to become important tools in clinical gene therapy.

In the case of HBV, published in vivo hydrodynamic transfection studies have shown that simultaneous delivery of HBV expression plasmids and HBV-specific synthetic shRNA (or ShRNA-expressing constructs) to the mouse liver can prevent the induction of HBV gene expression and replication [26-29]. But the possibility of the inhibition of HBV replication by lentiviral vector delivery of shRNA has not been systemically studied. Based on successful establishment of a murine model of acute HBV infection, in the present study, we selected two different RNAi target sites of HBV, adopted the hydrodynamics method, transduced mice hepatocytes with lentiviral vectors, and observed the effects of RNAi on the replication of HBV in animal experiments.

## Methods

### Plasmids and ShRNA

The vector of pTHBV2 containing the HBV genome plus a redundancy for the sequences between nt 1067 and 1996 of the HBV genome was donated by Dr. Mc Caffery (University of Iowa, Iowa City, Iowa, USA). Synthetic transcription shRNA template sequences are as follows: shRNA-1: forward: 5'-CCG GGA GGC GAG GGA GTT CTT CTT CTA GGG AAG CTT GCC TAG AGG AAG AGC TCC TTC GCC TCT TTT TG-3'; reverse: 5'-AAT TCA AAA AGA GGC GAA GGA GCT CTT CCT CTA GGC AAG CTT CCC TAG AAG AAG AAC TCC CTC GCC TC-3'.shRNA-2: forward: 5'-CCG GGA GGC GAA CAA ATG GCA CTA GTA AAC TGA GGA AGC TTG CTC AGT TTG CTA GTG TCA TTT GTT CTT TTT G-3'; reverse: 5'-AAT TCA AAA AGA ACA AAT GAC ACT AGC AAA CTG AGC AAG CTT CCT CAG TTT ACT AGT GCC ATT TGT TC-3'. Negative control shRNA(HBV-irrelevant shRNA): forward: 5'-CCG GGA GGC AAT CAG TCA ACA CCG TGC ATA TCC AGA AGC TTG TGG ATA TGC ACG GTG TTG ACT GAT TTT TTT G-3'; reverse: 5'-AAT TCA AAA AAA TCA GTC AAC ACC GTG CAT ATC CAC AAG CTT CTG GAT ATG CAC GGT GTT GAC TGA TT-3'. The expression plasmid pMKO.1-HBV-ShRNAs (shRNA-1, ShRNA-2 and Negative control shRNA) were constructed by subcloning above synthetic annealed duplexes between the AgeI and EcoR I sites of the plasmid of pMKO.1(Provided by Dr. William Hahn, Harvard University, USA) respectively. An approximately 300 bp small fragment, including U6 promoter and shRNA expression cassettes, was digested from pMKO.1-HBV-shRNA and pMKO.1-Negative control-shRNA by using Sal I enzyme. The recombinant lentiviral expression vector pWPT-HBV-shRNA-1, pWPT-HBV-shRNA-2 and pWPT-Negative control shRNA were constructed by inserting the 300 bp small fragment into the Sal I site of pWPT-GFP (provided by Dr. Trono, University of Geneva, Switzerland) [30].

### Lentivirus production and transduction

Recombinant lentivirus was generated from 293T cells (6 × 10^6 ^cells/100-mm plate) co-transfected with calcium phosphate precipitation of 3 plasmids: (1) pWPT-Negative control-shRNA or pWPT-HBV-shRNAs, and (2)pCMVΔR8.91, a plasmid expressing the HIV-1 gag/pol, tat, and rev genes required for efficient lentivirus production (gift from D. Trono), and (3) a plasmid expressing the vesicular stomatitis virus envelope glycoprotein (G), pLR-VSV-G (Invitrogen Corp. Carlsbad, CA, USA), at 15:15:7.5 μg/plate. Virus was collected and concentrated 500-fold by ultra-high speed centrifugation at 26,000 rpm, 4°C for 2 h, and stocks were stored frozen at -80°C until used as previously described[31]. Virus titer was determined by real-time quantitative reverse-transcriptase-PCR measuring copies of proviral DNA integrated into the genome of circulating murine mononuclear cells as recently described[32]. Replication-competent virions were confirmed absent from viral stocks by using extended marker rescue assays. After ultra-high speed centrifugation, the titers were adjusted to 10^9 ^transducing units (TU)/ml.

### Experimental animals

Yang and colleagues [33] reported that following hydrodynamic transfection of immunocompetent mice with an HBV plasmid, HBsAg-neutralizing antibodies were observed, starting at day 7. To avoid complications associated with neutralizing antibodies, experiments were carried out in NOD SCID mice lacking B and T lymphocytes. In addition, Park [34] reported that lentiviral vector transduction efficiency and transgene expression were significantly enhanced in adolescent (3 1/2 weeks of age) mice compared to older (7 weeks of age) mice. Thus, for all the *in vivo *experiments we used 3-4-week-old female immunocompromised NOD/LtSz-Prkdcscid/J (NOD SCID) mice (body weights 12-15 g) obtained from the National Resource Center for Mutant Mice, Model Animal Research Center of Nanjing University (Nanjing, China). And all the experiments were approved by the Ethics Committee of Nanjing Medical University and conducted in accordance with the guidelines approved by the China Association of Laboratory Animal Care. The pTHBV2 plasmid and lentiviral particles carrying shRNAs were delivered into mouse liver using the hydrodynamic tail vein injection method, a method that results in gene transfer into 5-40% of mouse hepatocytes[35,36]. Briefly, 12 μg pTHBV2 was dissolved in 1.6-2.0 ml serum-free medium or lentivirus. And then the medium was steadily and rapidly (no more than 4 - 6 seconds) injected with high pressure into the mouse tail vein using a 45-mm scalp needle. The mice were bled, and the sera were separated and examined for HBsAg, HBeAg or HBV DNA content on day 1, 4 or 7 after injection. Samples of mouse livers (5 mm × 6 mm size) were preserved in 4% paraformaldehyde (PFA) for histological analysis, while other samples (100-200 mg) were immediately frozen in liquid nitrogen.

Mice were randomly divided into four groups with six in each group. According to the materials they would receive by injection as follows: (i) Empty vector group: 12 μg pTHBV2 was dissolved in 1.6-2.0 ml serum-free medium. (ii) pWPT-HBV-shRNA-1 group: 12 μg pTHBV2 was dissolved in 1.6-2.0 ml pWPT-HBV-shRNA-1 lentivirus, co-injected. (iii) pWPT-HBV-shRNA-2 group: 12 μg pTHBV2 was dissolved in 1.6-2.0 ml pWPT-HBV-shRNA-2 lentivirus, co-injected. (iv) Negative control RNAi group: 12 μg pTHBV2 was dissolved in 1.6-2.0 ml pWPT-Negative control-shRNA lentivirus, co-injected.

### GFP expression in mouse liver

The pTHBV2 plasmid and lentiviral particles carrying GFP and shRNAs were delivered into mouse liver using the hydrodynamic tail vein injection method. Mouse livers were removed from the treated mice 4 or 7 days after lentiviral vectors injection respectively and fixed in formalin prior to embedding into paraffin. Liver sections (5 μm) were processed for the detection of green fluorescent protein (GFP) by incubating the tissues with a mouse anti-GFP monoclonal antibody (sc101525; Santa Cruz Biotechnology, Santa Cruz, CA, USA) at a dilution of 1:200. Protein was visualized with the chromogen substrate 3,3-diaminobenzidine (DAB). Hepatocytes were scored positive for GFP when clear brown staining of the cytoplasm could be identified. In each mouse, the GFP stained hepatocytes were determined separately by counting a total of 1500 hepatocytes in 20-30 randomly selected liver fields (three different liver lobes) and expressed as a percentage of all positive cells.

### Hepatitis B surface antigen (HBsAg) and hepatitis B e antigen(HBeAg) ELISA

Blood samples were obtained from the orbital sinus of experimental animals. Serum HBsAg was determined by a quantitative sandwich enzyme linked immunosorbent assay (ELISA) technique, using commercially available ELISA kits (Abbott). The level of HBeAg in the serum was measured by sandwich ELISA and quantified relative to a standard curve of serial dilutions of recombinant HBeAg (Abbott Laboratories, Abbott Park, IL USA). The relative concentration of HBeAg was represented by the absorbance value of specimens at 492 nm (A492).

### HBsAg and hepatitis B core antigen (HBcAg) expression in mouse liver

The formalin-fixed liver tissues samples from the treated mice were embedded in paraffin. Immunohistochemical staining for HBsAg and HBcAg was performed using the PV-6001 kit (Power Vision™ IHC Polymer Detection Reagent, USA) with specific antibodies against HBsAg (rabbit, OBT0990, Oxford Biotechnology Ltd., Oxford, UK) and HBcAg (rabbit, Boster Biological Technology, Wuhan, China). The immunological detection of these antigens was performed using the manufacturer's protocol.

### HBV DNA quantitative PCR analysis

HBV DNA was isolated from mice sera. Fluorescent real-time polymerase chain reaction (FQ-PCR) was performed to quantify HBV viral genomic DNA in the mice sera using an HBV diagnostic kit (PG Biotech, Ltd, Shenzheng, China) according to the kit's instructions. The primers specific for the detection of HBV S region were 5'-CCT CTT CAT CCT GCT GCT-3' and 5'-AAC TGA AAG CCA AAC AGT G-3'. The fluorescent probe was 5'-FAM-TCC CAT CCC ATC ATC CTG GGC TTT-TAMRA-3'. The reaction took place for 40 cycles in the iCycler iQ real-time PCR system (Bio-Rad Laboratories, USA). The inhibition ratio of HBV DNA was calculated according to the formula, which is [1-log(treated sample fluorescent intensity)/log(control fluorescent intensity)] × 100%.

### Quantitative real-time PCR assay

RT reactions were performed using the SuperScript First-Strand Synthesis System (Invitrogen Co. CA). To determine the number of cDNA molecules in the reverse transcribed samples, real-time PCR analyses were performed using the LightCycler system (Roche, IN). PCR was performed using 10 μl 2 × Master Mix SYBR Green I (Takara, Japan), 0.25 μl of each 5' and 3' primer, and 2 μl samples or H_2_O to a final volume of 20 μl. Samples were denatured at 94°C for 5 min. Amplification and fluorescence determination were carried out in three steps: denaturation at 94°C for 10 sec, annealing at 60°C for 15 sec, extension at 72°C for 20 sec; and at the end of extension detection of SYBR Green fluorescence, which reflects the amount of double-stranded DNA. The amplification cycle number was 35. To discriminate specific from nonspecific cDNA products, a melting curve was obtained at the end of each run. Products were denatured at 95°C for 3 sec, and the temperature was then decreased to 58°C for 15 sec and raised slowly from 58°C to 95°C using a temperature transition rate of 0.1°C/sec. Data were normalized with GAPDH levels in the samples. The primer sequences used for the real-time PCR were listed follows respectively: forward: 5'-CTG GGT GGG TGT TAA TTT GG-3', reverse: 5'-TAA GCT GGA GGA GTG CGA AT-3' for the HBV C region; and forward: 5'-ATG GAG AAC ATC ACA TCA GG-3', reverse: 5'-TTA AAT GTA TAC CCA AAG ACA AAA G-3' for the HBV S region. The primers for GAPDH (internal control) were: forward: 5'-AAC TTT GGC ATT GTG GAA GG-3', reverse: 5'-ACA CAT TGG GGG TAG GAA CA-3'. (synthesized by Sangon Biological Engineering Technology, Shanghai, China).

### Statistical analysis

All data are expressed as means ± S.D. The data were based on six animals per group *in vivo*. Comparison between groups was performed by an independent-samples *T *test. *P*-values of less than 0.05 were considered to be statistically significant.

## Results

### Selection of RNAi targets sites

**Figure 1:**
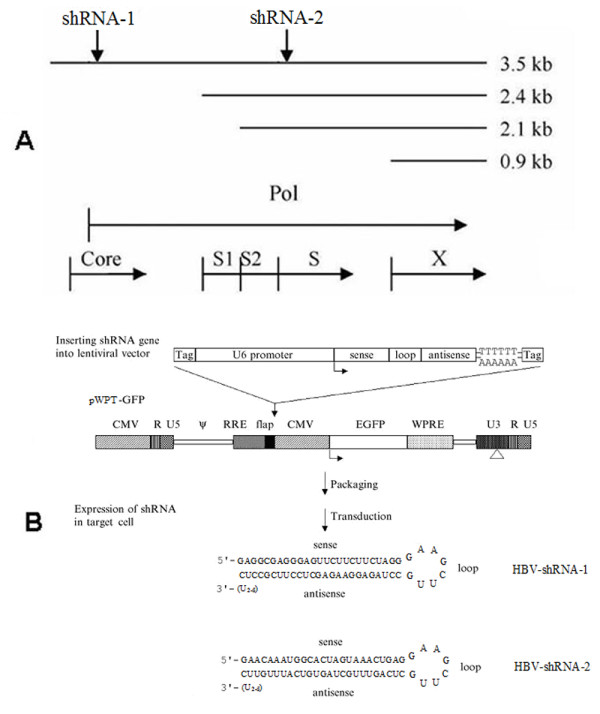
**Target site and vector information**. (A) Location of RNAi target sites. Downward arrows indicate the location of RNAi target sites within the four HBV transcripts. The 3.5-kb transcript is the pregenomic RNA that serves as the template for HBV viral DNA replication. The HBV open reading frames are shown below aligned with the HBV mRNAs. Pol: polymerase; Core: HBcAg; S1: large pre-surface antigen; S2: middle pre-surface antigen; S: HBsAg; X: X gene. (B) The construction schematic of lentiviral vector expressing shRNAs. The shRNA expression cassette, including the U6 pol III promoter, the sense and antisense sequence of the shRNA separated by a 8-base loop, and a terminator composed of six thymidines is inserted directly upstream of the CMV promoter of EGFP in the pWPT-GFP vector. Arrows indicate the orientation of transcription for a given gene. The putative HBVshRNA-1 and HBVshRNA-2 structures are shown at the bottom.

According to previous studies [27], two shRNA target sequences were chosen on the basis of their conservation among the major HBV genotypes adr, adw, ayr and ayw. In these cases, the sequences targeted overlapping reading frames of the virus such that multiple viral RNAs would be inhibited by one shRNA. Each shRNA targets the pregenomic RNA serving as the template for HBV genomic replication as well as the mRNA for the core antigen and the polymerase. Moreover, shRNA-1 targets the pregenomic RNA in the overlap region encoding the core antigen and the polymerase and shRNA-2 targets the HBV S-antigen mRNAs (Figure 1).

### Immunohistochemical detection of the expression of GFP in the liver tissues of mice

**Figure 2:**
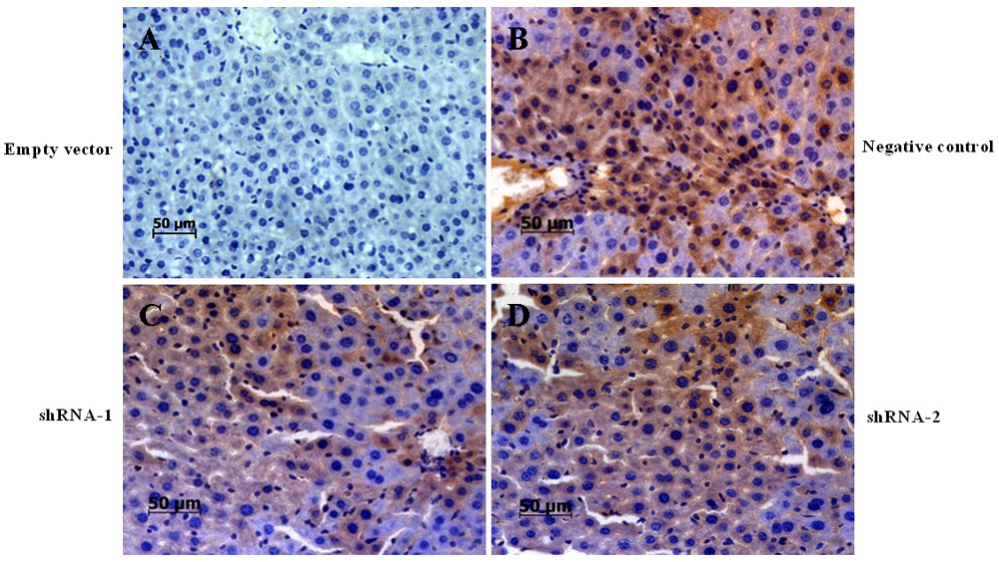
**Immunohistochemistry of the expression of GFP in liver tissue sections**. Lentiviral vectors were injected via tail vein by using the hydrodynamic method to the NOD SCID mice, as described in Materials and Methods. On day 4 after injection, animals were sacrificed and the livers were embedded and sectioned. Sections were stained immunohistochemically with anti-mouse GFP monoclonal antibody. The positive staining of GFP expression was detected in numerous liver cells in Negative control RNAi group (B), pWPT-HBV-shRNA-1 group (C), and pWPT-HBV-shRNA-2 group (D). A is section from empty vector group. (400× original magnification, scale bar 50 μm).

The mice were sacrificed on day 4 or 7 after lentiviral vectors hydrodynamic injection. Immunohistochemistry staining was performed to detect the expression of GFP in the liver tissues of mice treated with pWPT-GFP-ShRNAs lentivirus. Consistent with the expected transduction efficiency, each of the lentiviral vectors (pWPT-Negative control-shRNA or pWPT-HBV-shRNAs) treated mice exhibited about 11.3 ± 5.1% of positive staining cells of GFP on day 4 (Figure 2B, 2C, 2D), and showed similar results on day 7. While GFP was not detectable in the liver tissue sections in empty vector group(Figure 2A). These results showed that the lentiviral vectors we used in this study could transduce hepatocytes in vivo.

### Both HBsAg and HBeAg were inhibited by lentiviral transduced shRNA targeting HBV

To determine the effect and the minimum concentration of naked plasmids pTHBV2 for HBV expression in vivo, the mice were injected via the tail vein with 36 μg, 24 μg, 18 μg or 12 μg pTHBV2, and the levels of serum HBsAg were 112.21 ± 19.07 (S/N), 105.01 ± 21.66 (S/N), 103.14 ± 18.79 (S/N), and 100.54 ± 23.59 (S/N), respectively, after 1 day (S: the measure value of sample, N: the measure value of negative control sample; S/N≥2.0 as positive). Thus, we selected a pTHBV2 concentration of 12 μg (116.32 ± 19.73 ng/ml), as higher concentrations did not significantly increase serum HBsAg levels.

The contransfection model was established in which cotransfection with pTHBV2 and lentiviral particles pWPT-HBV-shRNA was used to test RNAi response for suppressing HBV antigen production in mice. All experiments were carried out in NOD SCID mice lacking B and T lymphocytes. Serum HBsAg and HBeAg levels were measured at day 1, day 4 and day 7.

**Figure 3:**
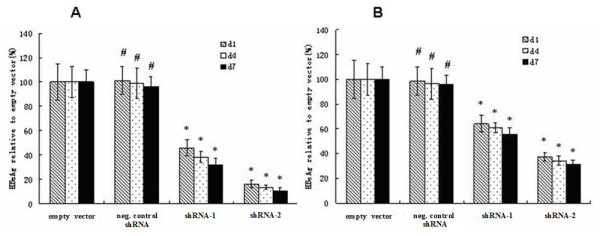
**Serum HBV antigens levels in HBV shRNA-treated mice**. (A) HBsAg levels in NOD SCID mouse sera were significantly reduced after treatment with the shRNA expression lentiviral vectors pWPT-HBV-shRNA-1 and shRNA-2. (B) The levels of HBeAg were significantly reduced by treatment with HBV shRNAs in mice. Inhibition of HBV antigens were specific and lasted at least 7 days (error bars indicate standard errors). The values showed the average of three experiments (means standard deviation). The mean value of empty control was defined as 100%. *P < 0.05 vs. the empty vector group. ^# ^*P *> 0.05 vs. the empty vector group.

The results of a timed study showed that there was no significant difference in serum HBsAg levels between empty vector group and Negative control shRNA group on days 1, 4 or 7. In contrast, serum HBsAg levels in pWPT-HBV-shRNA-1 group were significantly reduced (*P *< 0.05) on day 1, 4 and 7 by 54%, 61.8% and 68.1%, and levels in pWPT-HBV-shRNA-2 group were significantly reduced (*P *< 0.05) by 83.9%, 86.8% and 89.9% respectively (Figure 3A). These results convincingly demonstrated that pWPT-HBV-ShRNA reduced serum HBsAg in mice.

Similar significant results (*P *< 0.05) were obtained with the inhibition of serum HBeAg by lentivirus (Figure 3B). In pWPT-HBV-ShRNA-1 group, the inhibition of serum HBeAg levels was 35.7%, 39.1% and 44.3% on days 1, 4 and 7, respectively, while the inhibition of serum HBeAg levels in the pWPT-HBV-ShRNA-2 group at the same time point was 62.5%, 65.6% and 68.5%, respectively. The Negative control shRNA group exhibited no suppressive effect on HBeAg levels, demonstrating the specificity of shRNA effect.

### HBV RNA levels in liver tissue of mice were downregulated byusing HBV ShRNA

**Figure 4:**
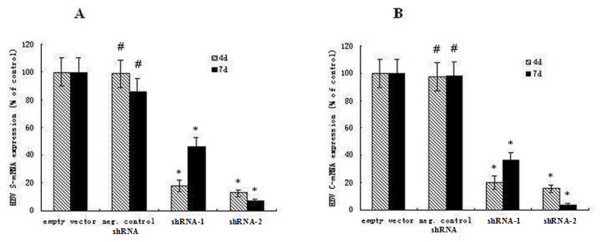
**The inhibitory effects on HBV RNA levels by transfection with HBV ShRNAs**. NOD SCID mice were cotransfected with 12 μg pTHBV2 and 1.6-2.0 ml pWPT-HBV- ShRNA-1, 2 or negative control shRNA. The mice were sacrificed 4 d and 7 d after cotransfection, and hepatocytes RNA was extracted. Each experiment was carried out in triplicate. (A) The total levels of HBV S-mRNA in NOD SCID mouse livers were significantly reduced on day 4 or 7 after treatment with pWPT- HBV-shRNA-1 and shRNA-2 lentivirus. (B) The total levels of HBV C-mRNA were significantly decreased on day 4 or 7 after treatment with pWPT-HBV-shRNAs in mice. (error bars indicate standard errors). The values shown were the average of three experiments (means standard deviation). The mean value of empty control was defined as 100%. * P < 0.05 vs. the empty vector group. ^# ^P > 0.05 vs. the empty vector group.

Real-time PCR analysis was performed to study the inhibitory effect of shRNA on viral RNA levels in the livers of mice. pWPT-HBV-ShRNA-1 treated mice exhibited significant mean decrease of 82.1% or 53.9% (*P *< 0.05, Figure 4A) in total levels of HBV S-mRNA, on day 4 or 7, respectively, compared to the untreated control mice. Whereas, pWPT-HBV-ShRNA-2 treated mice exhibited an average decrease of 87.5% or 92.9% (*P *< 0.05, Figure 4A) in HBV S-mRNA total levels, respectively. In addition, pWPT-HBV-ShRNA-1 or 2 treated mice exhibited an average decrease of 80.1%, 63.8% or 84.7%, 96.2% (*P *< 0.05, Figure 4B) in total levels of HBV C-mRNA on day 4 or 7 respectively. However, the Negative control RNAi group had no inhibitory effect.

### ShRNA-mediated HBV DNA inhibition in vivo

**Figure 5:**
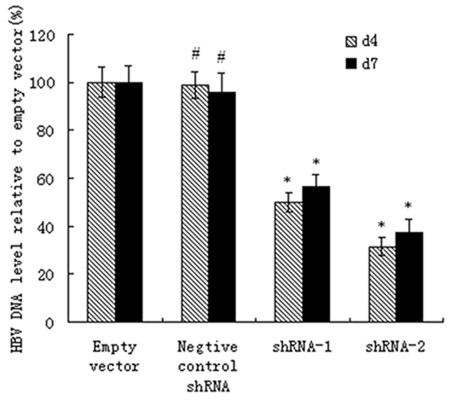
**Serum HBV DNA levels by HBV shRNAs in mice**. The levels of HBV DNA were significantly reduced by treatment with pWPT-HBV-shRNA lentivirus. The inhibition of HBV DNA was specific (error bars indicate standard errors). The mean value of empty control was defined as 100%. * P < 0.05 vs. the empty vector group. # P > 0.05 vs. the empty vector group.

To address this question, the mice sera were harvested and separated on day 4 and day 7 post-transfection. HBV DNA was isolated and FQ-PCR analysis was performed with total serum DNA from treated mice, using an HBV luciferase diagnostic kit (PG Biotech, China). Our results revealed that both the lentiviral vectors targeting HBV shRNA significantly suppressed (*P *< 0.05) HBV viral replication. The relative copy number of HBV DNA decreased significantly (*P *< 0.05) by 68.3% and 62.5% on day 4 and day 7 respectively in the mice treated by pWPT-HBV-ShRNA-2. While the inhibitory effect of pWPT-HBV-ShRNA-1 was 50.1% and 43.6% on day 4 and day 7, respectively (*P *< 0.05). The Negative control shRNA had no significant effect on HBV DNA titers (Figure 5).

### Immunohistochemical detection of decreased HBcAg and HBsAg expression in the livers of mice

**Figure 6:**
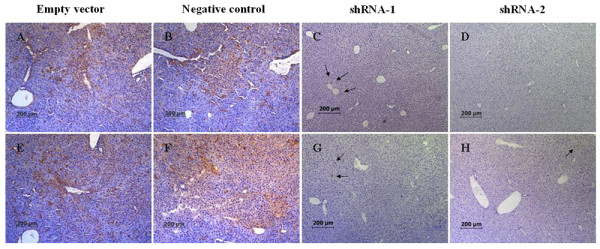
**Immunohistochemical staining for HBsAg (A-D) and HBcAg (E-H) in liver sections**. Treatment with pWPT-HBV-shRNA-1 lentivirus(C, G) or pWPT-HBV-shRNA-2 lentivirus (D, H) reduces the number of HBsAg or HBcAg-positive cells on day 7 respectively compared to the control. The staining was performed on tissue sections from three animals per group. Representative sections are shown (four sections per animals were stained and counted). Numerous HBsAg-positive and HBcAg-positive cells were seen in the empty vector group (A, E) and negative control RNAi group (B, F). No HBsAg-positive stained cells were observed in most fields in pWPT-HBV-shRNA-2 group(D). Some stained cells could be seen in pWPT-HBV-shRNA-1 or shRNA-2 groups(C, G, H) and these were indicated by arrows, but staining were much less intense than that in control groups (100× original magnification, scale bar 200 μm).

HBsAg, an envelope protein, is secreted into serum following infection. HBcAg, the nucleocapsid protein, is synthesized in infected cells and is required for HBV viral replication. To histologically determine the effects of ShRNAs on HBV antigens, immunohistochemical staining was conducted. At day 7, paraffin-fixed liver sections were prepared from the mice in different groups. Consistent with the expected transduction efficiency, 5.4 ± 1.3% and 5.1 ± 1.2% of cells stained for HBcAg and HBsAg in tissue sections from mice that only received the plasmid pTHBV2. Compared with the control group, the percentage of HBcAg-positive cells was significantly reduced by 99.1%, and HBsAg-stained cells were scarce in the livers of pWPT-HBV-ShRNA-2 group. Most fields had no stained cells, although there were rare hepatocytes with lightly stained cytoplasm. The percentage of HBcAg or HBsAg-stained hepatocytes in sections from pWPT-HBV-ShRNA-1 group was reduced by 92.0% and 84.5% respectively. In contrast, negative control shRNA group exhibited no inhibitory effect on the antigens (Figure 6). On the other hand, no staining was seen in sections from mice that did not receive pTHBV2 (result not shown). These results demonstrated that lentiviral vector mediated HBV RNAi can inhibit the production of HBV proteins.

## Discussion

HBV infection, especially chronic HBV infection, is one of the most common diseases threatening human health. However, current therapies for chronic HBV infection are limited. Due to the replication characteristics of HBV, RNAi has rapidly emerged as a potential gene therapeutic strategy for HBV infection. First, HBV is a DNA virus; its replication requires an intermediate pre-genomic RNA molecule for reverse transcription synthesis of viral DNA. Therefore, HBV is assumed to be susceptible to RNAi not only at the level of post-transcription, but also at the level of replication. Second, the HBV genome is small and its transcription units are overlapping, which allows the targeting of one site to inhibit multiple HBV mRNAs[37]. Third, the virus replicates almost exclusively in the liver, an organ that has proved to be accessible to in vivo gene therapy delivery techniques[38].

Nevertheless, chemically synthesized shRNA delivered to target cells is known has low and variable transfection efficiency and to be transient, only lasting 3-4 days[39], which limits the application of ShRNA in long-term gene silencing in mammalian systems. In order to address this issue, different viral gene delivery vectors have been tested. Several studies indicated that the adenovirus or retrovirus mediated RNAi delivery system was an extremely effective and long-term approach to express shRNA targeted to HBV and could suppress ongoing viral gene expression and replication in vitro or in vivo[21,40]. However, as adenoviral vectors are episomal, they are not subject to persistent effects, but are lost upon cell division, and being a common human pathogen, a large segment of the general population has pre-existing immunity against adenoviral proteins. Since retroviruses require cell division for integration they are inefficient in transducing genes into quiescent hepatocytes. In this study we developed an appropriate lentivirus mediated shRNA delivery strategy to generate RNAi. Lentiviral vectors are able to transduce quiescent cells efficiently, including brain, liver, muscle, and hematopoietic stem cells [41-43] with no apparent requirement for cell proliferation. Some groups [44-46] have reported that lentiviral vectors can efficiently deliver shRNA expression cassettes into a variety of cells with sustained long-term expression and without observable toxicity[47,48]. Although there are lingering biosafety concerns regarding HIV-1 based lentiviral vectors, the current third-generation replication-defective and self-inactivating (SIN) lentiviral vectors have minimized the potential risk of generating replication-competent helper virus[31]. In the present study, we used pWPT-GFP, a typical third-generation replication-defective SIN vector, as the transfer vector for shRNA. To our knowledge, this is the first report of the use of this lentivirus expression system containing a GFP report gene and U6 small nuclear RNA promoter to deliver shRNA into mouse livers to silence HBV genes.

For a long time, largely due to the narrow host restriction of HBV and the deficiency of an ideal HBV animal model, the investigation of HBV infection in vivo was extremely limited. A hydrodynamics mouse model was reported [33] that alleviates some of these experimental constraints and permits HBV to replicate for a period of time. This type of convenient animal model has been adopted in some studies in which shRNA was used against HBV[27,33,35,36,49]. In the present study, we detected HBV DNA production and HBsAg secretion after hydrodynamically injecting NOD SCID mice with the HBV genomic plasmid pTHBV2 via the tail vein.

The design of shRNA is very important in gene silencing. Based on previous studies[27,49], two shRNA duplexes targeting the overlap region encoding the core antigen and the polymerase (POL)/surface antigen region (HBsAg) of the HBV genome were selected. That is, they both target the 3.5-kb transcript, which not only serves for translation of the core protein/HBeAg and polymerase-reverse transcriptase but also represents the template for reverse transcription. In this study, four separate line of evidence establish that lentivirus mediated delivery of shRNA to the liver of mice is capable of substantially inhibiting HBV replication and expression in vivo. Quantitative sandwich ELISA revealed that HBsAg and HBeAg secreted in mice sera had been significantly reduced, consistent with the substantially decreased number of cells staining for HBcAg or HBsAg (intensity of staining were also decreased) relative to the controls. Transduction with lentiviral vector mediated shRNA resulted in a parallel reduction of viral DNA synthesis. In addition, real time PCR analysis demonstrated that the HBV S-mRNA and C-mRNA levels were significantly reduced in mouse liver.

The present study showed that shRNA-1 also reduced the levels of HBV S-mRNA and serum HBsAg, even though it did not directly target the surface antigen gene sequence in the genome. This result may be due to the inhibiting effects of the shRNA on the 3.5-kb pregenomic RNA, reverse transcription template for synthesizing HBV DNA replication intermediates. Alternatively this result suggests the possibility of some sequence-independent mechanisms which need to be further studied while ShRNA exerts its anti-viral effect. McCaffrey *et al*[27], Wu *et al*[28], and Ying *et al*. [29]also obtained similar results. On the other hand, similar to results reported by Uprichard *et al*[21], we also observed that shRNA mediated suppression of serum HBeAg was not as dramatic as the suppression of serum HBsAg. It was suggested that the RNAi resistance of this subset does not appear to be due to secondary structure masking of a particular target sequences; rather, it appears to be due to a more global protection of a subset of the HBeAg mRNA within RNA-protein complexes, perhaps polyribosome complexes on the rough endoplasmic reticulum. In addition, our results were similar to those reported by McCaffrey et al[27] and Ying et al[29], except that in our work, the inhibition ratio of HBV expression and replication were not immediately reduced. Instead it increased slightly at day 4 and 7 after transduction of shRNA. It was possibly because the shRNA we delivered were integrated into the host cell genome, and could sustain long-term suppression effects. Meanwhile, the immunocompromised NOD SCID mice, which lack functional T cells, B cells, and natural killer (NK) cells, were selected in this experiment, excluding the influence of complications associated with neutralizing antibodies, yielding more consistent results.

It is undeniable that using lentiviral vectors expression system for therapeutic application also has its limitations. For example, potential insertional mutagenesis may occur using an integrating viral vector or potential liver damage due to vector-related toxic side effects that may occur. Besides, overexpression of hairpins from a U6 promoter within AAV vectors has been shown to be toxic. Stable integration of similar cassettes, such as the system which were used in this study, potentially had any undesirable effects.

While attempting to develop a different mechanism of combating HBV infection, Chen *et al*[26] reported that a combination of shRNA with lamivudine provided a greater inhibition of HBV replication. In summary, although much work remains before making the transition from using shRNA in a mouse model to the administration of shRNA in clinical therapy, our study has shown that the long-term expression of shRNA mediated by a lentiviral gene delivery vector might be applicable as a novel effective gene therapeutic strategy. It could be used in combination therapy with the nucleoside analogs for the prevention and treatment of chronic HBV infection, and for other persistent infections such as HCV and HIV. The therapeutic potential of shRNAs remains promising.

## Conclusion

This study revealed that lentivirus-based RNAi is capable of inhibiting HBV replication and expression *in vivo *and thus may constitute a potential therapeutic strategy for HBV and other viral infections and provide new clues for prophylactic vaccine development in the near future.

## Competing interests

The authors declare that they have no competing interests.

## Authors' contributions

LD and GL: Carried out the molecular genetic studies, participated in the sequence alignment and drafted the manuscript. LX and AY: Carried out the immunoassays. YG: Participated in the sequence alignment. WY: Participated in the design of the study and performed the statistical analysis. XW and BS: Conceived of the study, and participated in its design and coordination and helped to draft the manuscript. All authors read and approved the final manuscript.

## Pre-publication history

The pre-publication history for this paper can be accessed here:

http://www.biomedcentral.com/1471-230X/9/73/prepub
